# Smoking induced salivary microbiome dysbiosis and is correlated with lipid biomarkers

**DOI:** 10.1186/s12903-024-04340-4

**Published:** 2024-05-25

**Authors:** Layla I. Mohammed, Rozaimi Razali, Zain Zaki Zakaria, Fatiha M. Benslimane, Farhan Cyprian, Maha Al-Asmakh

**Affiliations:** 1 Department of Biomedical Sciences, College of Health Science, QU-Health, Qatar University, PO Box 2713, Doha, Qatar; 2 Biomedical Research Center, Qatar University, Doha, 2713 Qatar; 3 The KINDI Center for Computing Research, College of Engineering, Qatar University, Doha, Qatar; 4 Medical and Health Sciences Office, QU-Health, Qatar University, PO Box 2713, Doha, Qatar; 5 Basic Medical Science Department, College of Medicine-QU Health, Qatar University, Doha, 2713 Qatar

**Keywords:** Oral microbiome, Smoking, 16s rRNA gene sequencing, Saliva, Qatar

## Abstract

**Background:**

The oral microbiome plays an essential role in maintaining oral homeostasis and health; smoking significantly affects it, leading to microbial dysbiosis. The study aims to investigate changes in the oral microbiome composition of smokers in the Qatari population and establish a correlation with lipid biomarkers.

**Methods:**

The oral microbiota was profiled from saliva samples of 200 smokers and 100 non-smokers in the Qatari population, and 16s rRNA V3-V4 region were sequenced using the Illumina MiSeq platform. The operational taxonomic units (OTUs) were clustered using QIIME and the statistical analysis was performed by R.

**Results:**

Non-smokers exhibited a more diverse microbiome, with significant alpha and beta diversity differences between the non-smoker and smoker groups. Smokers had a higher abundance of Firmicutes, Bacteroidota, Actinobacteriota, Patescibacteria, and Proteobacteria at the phylum level and of *Streptococcus, Prevotella, Veillonella, TM7x*, and *Porphyromonas* at the genus level. In contrast, non-smokers had more Bacteroidota, Firmicutes, Proteobacteria, Fusobacteriota, and Patescibacteria at the phylum level, and *Prevotella, Streptococcus, Veillonella, Porphromonas*, and *Neisseria* at the genus level. Notably, *Streptococcus* was significantly positively correlated with LDL and negatively correlated with HDL. Additionally, *Streptococcus salivarius*, within the genus *Streptococcus*, was substantially more abundant in smokers.

**Conclusion:**

This study highlights the significant influence of smoking on the composition of the oral microbiome by enriching anaerobic microbes and depleting aerobic microbes. Moreover, the observed correlation between *Streptococcus* abundance and the lipid biomarkers suggests a potential link between smokers-induced salivary microbiome dysbiosis and lipid metabolism. Understanding the impact of smoking on altering the oral microbiome composition and its correlation with chemistry tests is essential for developing targeted interventions and strategies to improve oral health and reduce the risk of diseases.

## Background

A healthy oral microbiome is colonized by 50 to 100 million bacteria belonging to approximately 700 distinct species, rendering it the second most abundant and diverse microbiome in the human body following the gut [1, 2]. According to multiple studies, the phyla predominantly found in the oral microbiome are Bacteroidetes, Firmicutes, Proteobacteria, Actinobacteria, and Fusobacteria [3, 4]. The oral cavity is a dynamic ecosystem, which provides an environment suitable for microbial colonization with a pH range of 6.5 to 7.5 and an average temperature of 37 °C in saliva [5]. It is an open system influenced by several factors, including diet, lifestyle, and environmental exposures. One notable exposure is smoking, which has been shown to significantly impact the oral cavity by introducing it to several toxins, leading to a disturbance known as microbial dysbiosis.

Smoking is a widespread practice that affects almost every organ system in the body [6] Approximately 1.1 billion individuals are actively smoking, and 1.9 billion are passive smokers [7]. It is a global health concern associated with increased risk of certain diseases such as dental caries, periodontitis, oral diseases, cardiovascular diseases (CVD), chronic obstructive pulmonary disease (COPD), and various types of cancer. Smoking-induced microbial dysbiosis can foster anaerobic conditions, promoting the proliferation of pathogenic bacteria due to reduced oxygen availability, ultimately culminating in disease [8, 9].

The impact of smoking on the oral microbiome has been previously studied, and the results have been inconsistent due to various factors. These include a limited number of participants, variation in sampling sites, and differences in laboratory methodologies, some of which may constrain bacterial profiling [8, 10–15]. Smoking has been linked to shifts in the oral bacterial genera and has demonstrated correlations with inflammation and carcinogenesis-associated hormones and cytokines [11]. Further investigations have indicated alterations in the oral microbiome of smokers, resulting in an environment favoring anaerobes [8, 13]. While the separate influences of smoking and microbial dysbiosis on diseases have been studied, their combined effect remains an enigma.

This study aims to advance our understanding of the impact of cigarette smoking on oral microbial composition and how this is correlated with metabolic syndrome biomarkers. Unfortunately, Qatar is among the nations with the highest incidence of CVD and metabolic syndromes [16]. The rapid transition to a Westernized lifestyle, characterized by sedentary living, calorie-rich diet consumption, and urbanization, has resulted in a higher incidence of metabolic diseases (obesity, CVD, diabetes, hypertension) [17]. This study aims to investigate the effect of smoking on oral microbiome dysbiosis and its correlation with metabolic syndrome biomarkers in the Qatari population.

## Methods

### Study cohort

Qatar Biobank (QBB) is a large-scale health initiative to provide biological samples and population data to scientists for research support to guide healthcare strategies for effective prevention of diseases and the development of new treatments (https://www.qatarbiobank.org.qa/). In the current study, 300 subjects were randomly selected from QBB, irrespective of their age and health status. 200 subjects self-reported being smokers, and 100 were non-smokers. Information on their general health status, disease history, and medications was collected on a designed questionnaire. Data for clinical chemistry was obtained from QBB. Saliva samples (500 µL) were collected from the participants by spitting directly into sterile tubes and immediately frozen at -80^o^C. These saliva samples were used for microbiome analysis. All the participants signed an informed consent form to use their data and biological samples as anonymous volunteers. The study was approved by the QBB Institutional Review Board (IRB) (IRB-QBB-2019-001) and Qatar University IRB (QU-IRB 1390-E/20). During the laboratory and data analysis, the research team followed research ethics, morals and biosafety guidelines according to the regulation by Qatar University and maintained participants’ anonymity [18].

### Saliva for microbiome analysis

Saliva samples were transported on ice from QBB to Qatar University. 200 µL of saliva samples were subjected to DNA extraction using a commercially available DNA extraction kit (QIAamp DNA Mini Kit, 51,306, MD, USA) according to the manufacturer’s instructions. The concentration and quality of the DNA were measured using Qubit-4 (Life Technologies, Carlsbad, California, US) and NanoDrop-2000 (Thermo Fisher Scientific, Waltham, Massachusetts, US). DNA extraction was sent to ABM Company (Richmond, Canada) for 16s rDNA metagenomics sequencing. The samples were prepped using the 16s rDNA Amplicon Sequencing pipeline. The quality of these libraries was assessed by Agilent Bioanalyzer 2100 and qPCR. The samples were pooled at equal concentrations. Paired-end sequencing was performed on the Hiseq 4000. A total of 12.3 million paired-end reads were obtained on demultiplexing using the provided adaptors.

### Bioinformatics analysis

The MiSeq run generated output in the form of FASTQ files, which were processed using the MiSeq Reporter software. The subsequent bioinformatics analysis utilized QIIME2 [19], employing various plugins for tasks such as quality control, filtering, assembly, OTU clustering, and taxonomy assignment. Since the dataset was already demultiplexed by sample, we imported using the sequences into qiime via the qiime import module with --type SampleData[PairedEndSequencesWithQuality] and --input-format PairedEndFastqManifestPhred33V2. Subsequently, sequence trimming to a uniform length and removal of non-biological sequences were performed using the QIIME cutadapt trim-paired command, where we used the default options except for --p-cores 31 and --p-error-rate 0.01. To group similar sequences, the denoising method was implemented using the qiime dada2 denoise-paired module using the options --p-trim-left-f 13 --p-trim-left-r 13 --p-trunc-len-f 300 --p-trunc-len-r 300 and --p-n-threads 31. We opted for inferring exact sequence variants, known as amplicon sequence variants (ASVs), which represent unique biological sequences. This allows for more precise taxonomic assignment and diversity analysis compared to the older OTU clustering method. For OTU clustering, close-reference clustering was executed with the QIIME vsearch cluster-features-closed-reference, specifying a percent identity of 0.97. Taxonomy assignment for the bacterial 16 S rRNA marker gene was accomplished using the QIIME feature-classifier classify-consensus-search, with parameters set at a percent identity of 0.9 and a query coverage of 0.829. The Silva 138 database, containing 99% OTUs full-length sequences, was employed as the reference database https://www.arb-silva.de/documentation/release-138/ [20].

### Statistical analysis

The statistical analysis and plots were performed using R version 4.3.1 (2023). Wilcoxon test was performed to compare the mean differences in plasma biochemistry and demographic characteristics between the study groups. Plots were done by using the ggplot2 package, version 3.4.3, and for significance in plots, the ggsignif package, version 0.6.4. Samples with insufficient reads were excluded, and the microbial relative abundance lower than 10% were replaced with a 0. In total 244 samples were included in the analyses. Three different alpha diversity metrics (sobs, Shannon, and Simpson) and beta diversity (Bray – Curtis dissimilarity) were calculated using the vegan package 2.6.4 [21]. The significance for beta diversity was calculated using the adinos2 function (PER-MANOVA) from the vegan package. Wilcoxon test was performed for statistical significance for microbial data, and Benjamini-Hochberg (BH) method was applied for the *p*-value adjustment. A *p*-value less than 0.05 was considered statistically significant. Spearman’s rank correlation coefficient was applied to measure the correlation between the microbial taxa and participants’ data.

## Results

### Characteristics of the study participants

Our study included 300 samples from the Qatari population who completed a smoking and health status questionnaire. The clinical characteristics of the sample are summarized in Table 1. As reported in Tables 1 and 200 (66.66%) were smokers and 100 (33.33%) were non-smokers. Notably, among the smokers, 17 individuals reported to be diabetic, 46 reported to have high cholesterol and 24 reported to have high blood pressure.

Smokers were significantly older with higher triglycerides, higher low-density lipoprotein (LDL), lower high-density lipoprotein (HDL), and a lower ratio of forced expiratory volume in 1 s (FEV1) versus forced vital capacity (FVC).

**Table 1:** Participant characteristics

	Non Smokers *n* = 100	Smokers *n* = 200	*p*-value
Age	32.63 ± 9.20	35.44 ± 9.85	**0.03**
BMI (Kg/m^2^)	28. 52 ± 5.18	27.84 ± 5.43	0.3
Smoking Duration	-	13.88 ± 11.80	-
Systolic bp (mm/Hg)	110.04 ± 13.63	110.97 ± 12.62	0.38
Diastolic bp (mm/Hg)	65.94 ± 8.63	66.99 ± 9.76	0.2741
FEV1 (L)	2.94 ± 0.8	2.74 ± 1.16	0.6876
FVC (L)	3.53 ± 1.0	3.37 ± 1.43	0.7088
FEV1/FVC	0.84 ± 0.08	0.73 ± 0.26	**0.000771**
Cholesterol (mmol/L)	4.83 ± 0.83	5.11 ± 1.16	0.06784
Triglycerides (mmol/L)	1.17 ± 0.66	1.37 ± 0.94	**0.033**
HDL (mmol/L)	1.45 ± 0.37	1.28 ± 0.38	**0.000639**
LDL (mmol/L)	2.85 ± 0.77	3.23 ± 1.07	**0.0045**
Glucose (mmol/L)	4.9 ± 0.52	5.01 ± 1.4	0.1668

BMI: Body Mass Index. bp: Blood pressure. FEV1: Forced Expiratory Volume in 1 s. FVC: Forced Vital Capacity. HDL: High density lipoprotein. LDL: Low density lipoprotein. *p*-value < 0.05 is significant in bold.

### Bacterial diversity and community of salivary microbiota

**Fig. 1:**
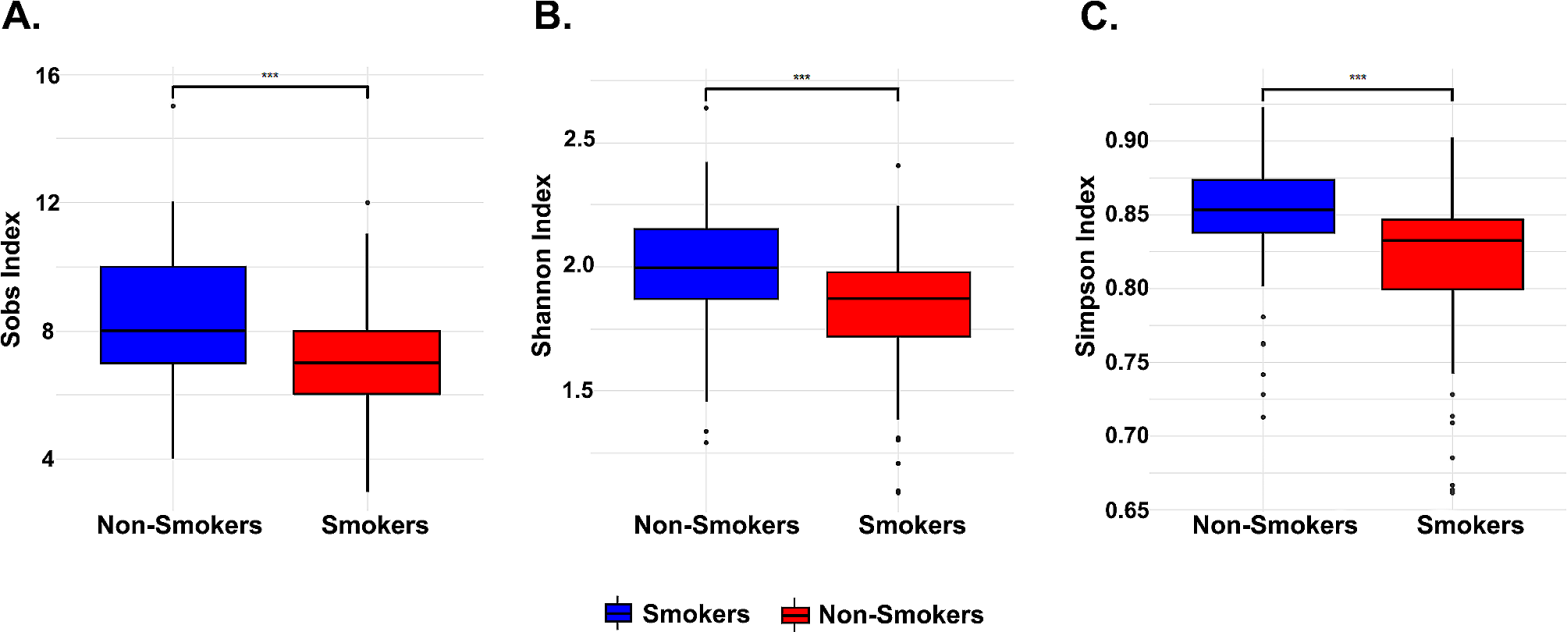
Boxplots of alpha diversity metrics. **(A)** Observed species richness **(B)** Simpson Index **(C)** Shannon index. Red boxplots represent the smokers and blue represent the controls. Statistically significant differences analysis was with Wilcoxon test

To investigate the effect of smoking on salivary microbiota diversity, we analyzed three alpha metrics on the smoker and non-smoker groups: species richness, Simpson index, and Shannon index. Non-smokers had a higher alpha diversity using three alpha diversity metrics (Sobs, Simpson, and Shannon indexes) (Fig. 1A, B & C). Alpha diversity represents microbiome diversity per sample. Non-smokers had a significantly higher species richness than smokers (Fig. 1A). The salivary microbiome was significantly richer and more diverse in the non-smokers as described by the Simpson index, which considers the number of species and the relative abundance (Fig. 1B). The Shannon index considers the number and the abundance of species found in a sample together (Fig. 1C). The more diverse the species within a sample, the higher the Shannon index; our results show that the non-smoker group is significantly more diverse. Table 2 provides an overview detailing each metric’s mean, standard deviation, and *p*-values.

**Table 2:** Alpha diversity. Represents the mean, standard deviation, and *p*-value of each alpha diversity metric

Alpha diversity	Non-Smokers	Smokers	*p*-value
Sobs	8.37 ± 1.81	7.15 ± 1.72	< 2.2E-16
Simpson	0.85 ± 0.04	0.82 ± 0.05	< 2.2E-16
Shannon	2.00 ± 0.23	1.82 ± 0.26	< 2.2E-16

**Fig. 2:**
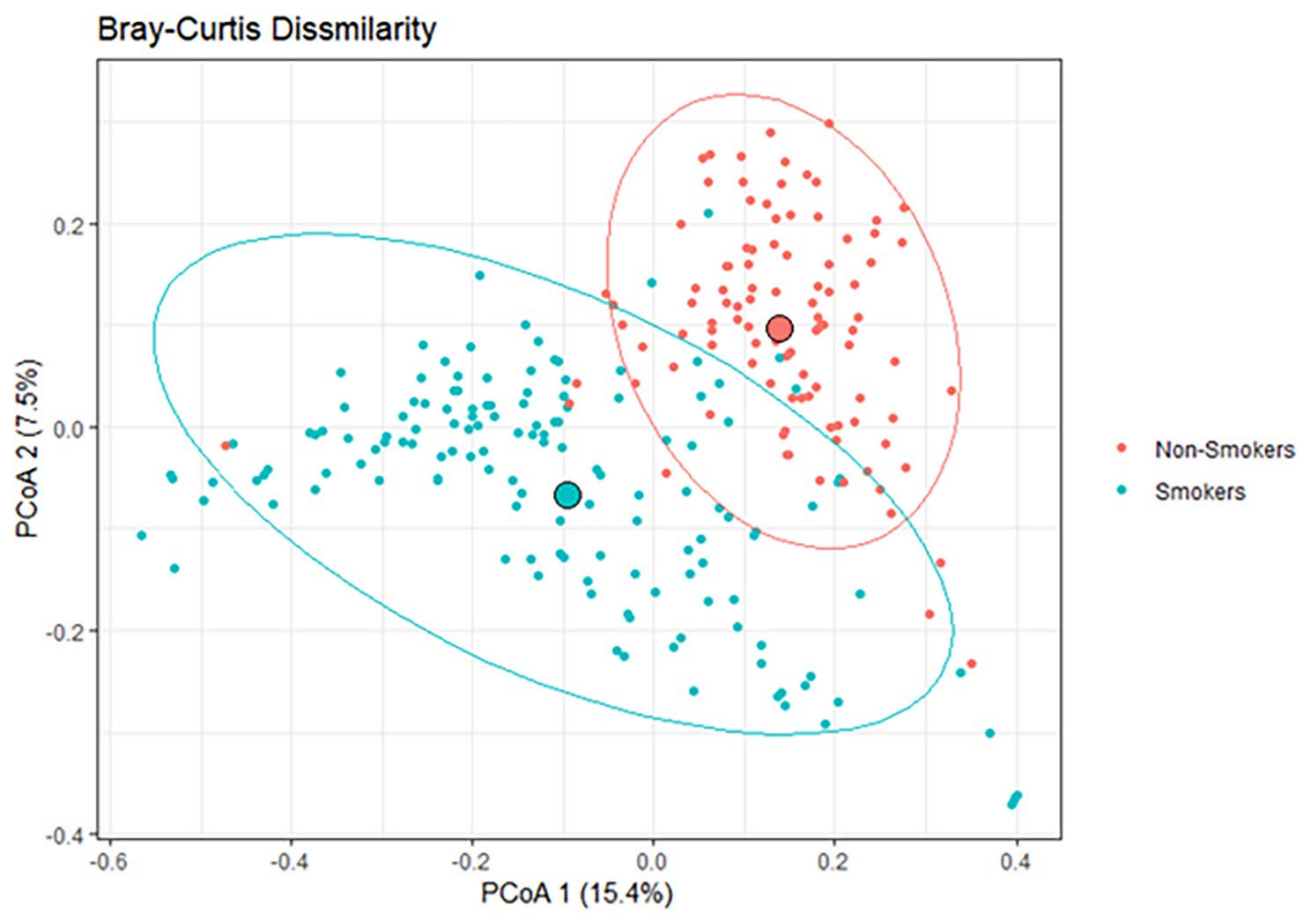
Beta diversity. Principal coordinates analysis (PCoA) of the distance matrix generated using Bray-Curtis Dissimilarity. Red is the control and blue are the smokers

To analyze beta diversity (diversity of microbiome within a community), we conducted Bray. Curtis dissimilarity, as shown in (Fig. 2.) Principal coordinates analysis (PCoA) was used to visualize the clustering of the similarities between non-smokers and smokers. Bray-Curtis compares the abundance of each species between non-smokers and smokers to give a parameter between 0 and 1. This metric quantifies the difference in abundance between the difference between samples and to visualize the two samples are similar. There is a significant difference between the non-smokers and smokers’ groups (PER-MANOVA, *p*-value < 0.001).

### Visualization of the taxonomic relative abundance

**Fig. 3:**
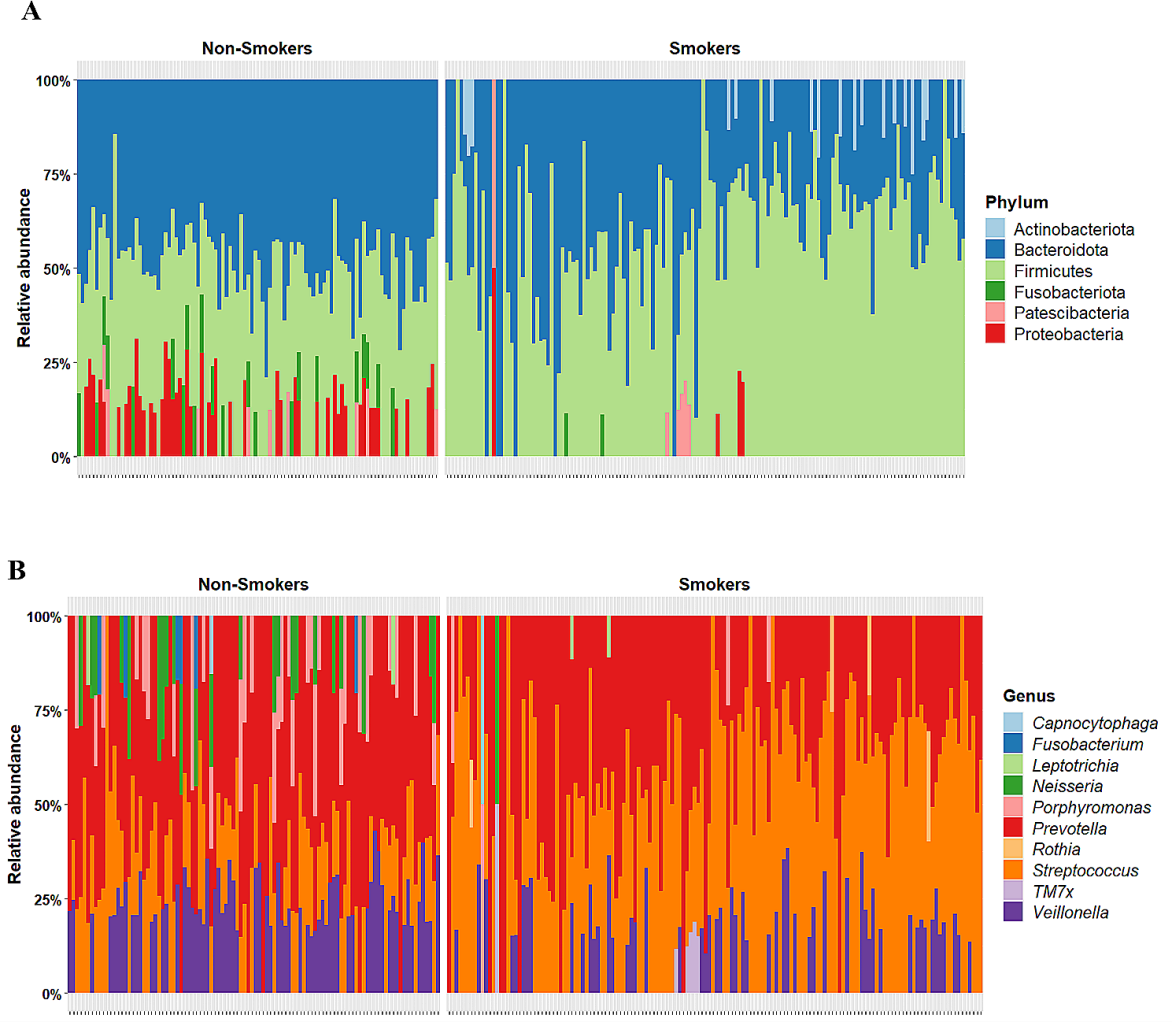
Relative abundance of saliva microbiome ≥ 10%. **(A)** Relative abundance per sample at the phylum level. **(B)** Relative abundance per sample at the genus level

The relative abundance of phylum and genus were generated from taxonomy table. After filtration, the number of the non-smoker group remained at 100, and the smoker group declined to 144 samples. Relative abundant stacked bar charts were generated for visualization to compare non-smokers and smokers (Fig. 3). The mean of the 5 dominant phyla in the non-smoker group were, Bacteroidota (41.46%), Firmicutes (33.2%), Proteobacteria (7.29%), Fusobacteriota (2.79), and Patescibacteria (1.22%) covering on average 86% of the non-smokers microbiome. The mean of the 5 abundant phyla from the smokers group were Firmicutes (53.42%), Bacteroidota (36.44%), Actinobacteriota (1.92%), Patescibacteria (0.86%), and Proteobacteria (0.64%), covering on average 93% of the smokers Qatari salivary microbiome. At the genus level the top 5 genera of their mean in the non-smoker group were *Prevotella* (29.22%), *Streptococcus* (12.39%), *Veillonella* (10.48%), *Porphyromonas* (4.46%), and *Neisseria* (3.32%) composing approximately of 60% of the genus level of the non-smokers group. However, the top 5 mean of the genera in the smoker’s group were *Streptococcus* (38.19%), *Prevotella* (33.37%), *Veillonella* (6.73%), *TM7x* (0.8%), and *Porphyromonas* (0.71%) making 80% of the genera in the smoker’s salivary microbiome.

### Analysis

**Fig. 4:**
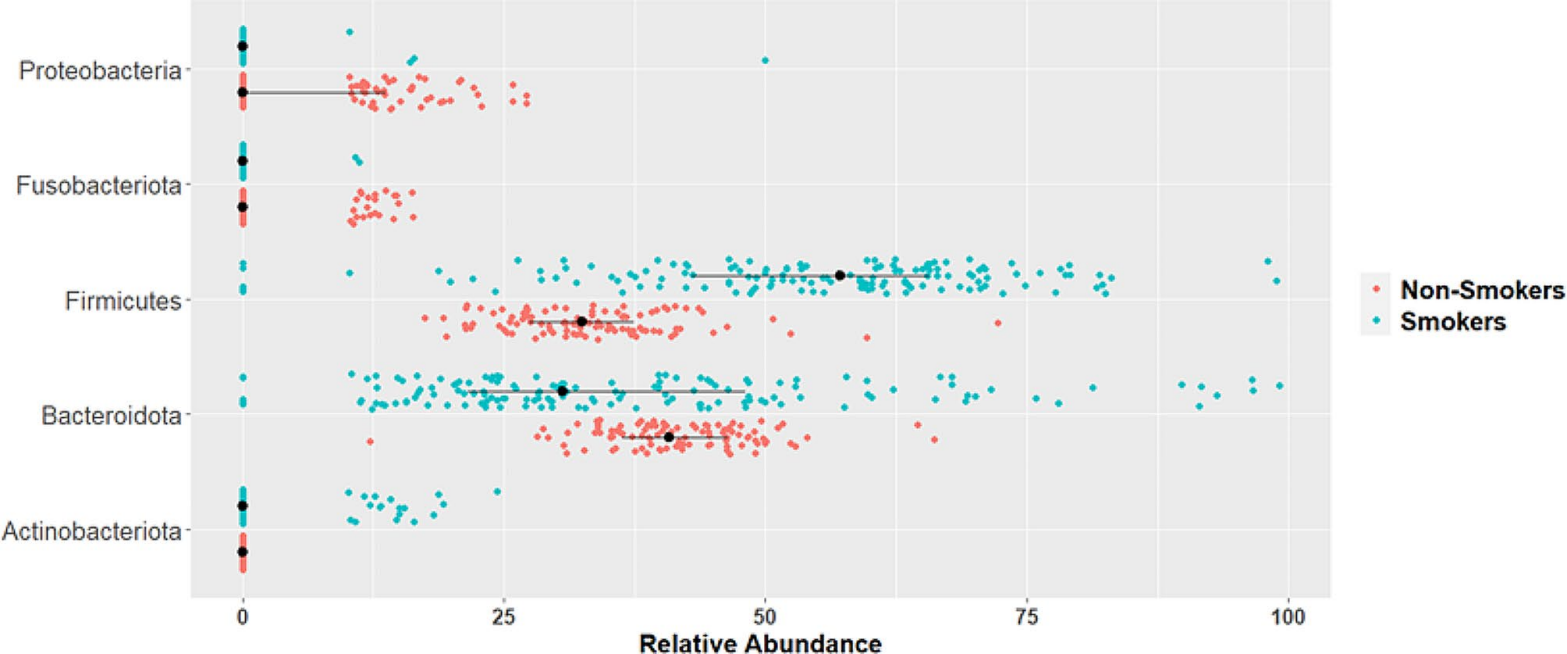
Visualization of the significant phyla between smokers and non-smokers. Wilcoxon test was performed *p*-value < 0.05

The assessment of significance between smokers and non-smokers is represented in Table 3. It includes the fold change, *p*-value, and adjusted *p*-value at the phylum level. There was a 1.61-fold significant increase in Firmicutes in the smokers group. In contrast, there was a significant decline in the levels of Proteobacteria, Fusobacteria, and Bacteriodota with 11.34, 18.33, and 1.14 fold changes, respectively, in smokers. Actinobacteriota was not detected in the non-smoker samples with a cutoff value abundance equal to or higher than 10% (Fig. 4) displays the significant phylum.

**Table 3:** Summary of phylum level mean relative abundance in non-smokers and smokers with fold change. The asterisk (*) indicates the significant values

Phylum	Non-Smokers	Smokers	Fold Change	*p*-value	*p*-adjust (BH)
Firmicutes	33.20%	53.41%	1.61	1.86E-20 *	1.11E-19 *
Proteobacteria	7.29%	0.64%	-11.34	4.93E-16 *	1.48E-15 *
Fusobacteriota	2.79%	0.15%	-18.33	9.05E-08 *	1.81E-07 *
Bacteroidota	41.46%	36.44%	-1.14	7.25E-05 *	1.09E-04 *
Actinobacteriota	0%	1.92%	-	1.64E-05 *	0.000197 *
Patescibacteria	1.22%	0.86%	-1.42	1.35E-01	1.35E-01

**Fig. 5:**
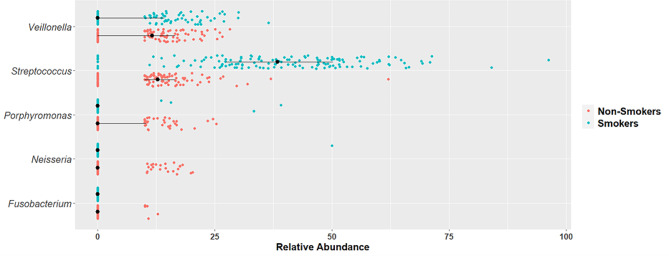
Visualization of the significant genera between smokers and non-smokers. Wilcoxon test was performed *p*-value < 0.05

The differences of the salivary microbiome on the genus level were identified. The relative abundance of *Streptococcus* increased by three folds in smokers. A significant 6.32, 9.57, and 1.57 fold reduction in the genus *Porphyromonas*, *Neisseria*, and *Veillonella*, respectively, was detected in smokers in comparison to non-smokers. *Fusobacterium* was not detected in smokers; however, 0.65% was detected in non-smokers. Table 4. Summarizing these results, Fig. 5 provides a visualization of the significant genera.

**Table 4:** Summary of genus level mean relative abundance in non-smokers and smokers with fold change. The asterisk (*) indicates the significant values

Genus	Non-Smokers	Smokers	Fold Change	*p*.value	*p*.adjust (BH)
*Streptococcus*	12.40%	39.20%	3.08	5.53E-25 *	5.53E-24 *
*Porphyromonas*	4.46%	0.71%	-6.32	1.56E-09 *	7.82E-09 *
*Neisseria*	3.32%	0.35%	-9.57	1.28E-08 *	4.26E-08 *
*Veillonella*	10.48%	6.73%	-1.56	8.84E-04 *	2.21E-03 *
*Fusobacterium*	0.65%	0%	-	3.01E-03 *	6.03E-03 *
*TM7x*	0%	0.80%	-	3.95E-02 *	6.58E-02
*Rothia*	0%	0.47%	-	9.44E-02	1.35E-01
*Leptotrichia*	0.23%	0.15%	-1.49	7.03E-01	8.79E-01
*Capnocytophaga*	0.10%	0.23%	2.30	8.04E-01	8.93E-01
*Prevotella*	29.22%	33.37%	1.14	9.92E-01	9.92E-01

As it has been mentioned above, *Streptococcus* is prevalent in smokers, our analysis also revealed the significance of *Streptococcus salivarius* (*S. salivarius*), at the species level, with a mean relative abundance of 14% in smokers and no detection in non-smokers. During analysis a potential novel *Streptococcus* species and Fusobacterium species were identified. There was a notable doubling in the presence of the novel *Streptococcus; __* among smokers , while the novel *Fusobacterium; __* species was detected in non-smokers with a relative abundance of 0.5%.

### Correlation analysis

**Fig. 6:**
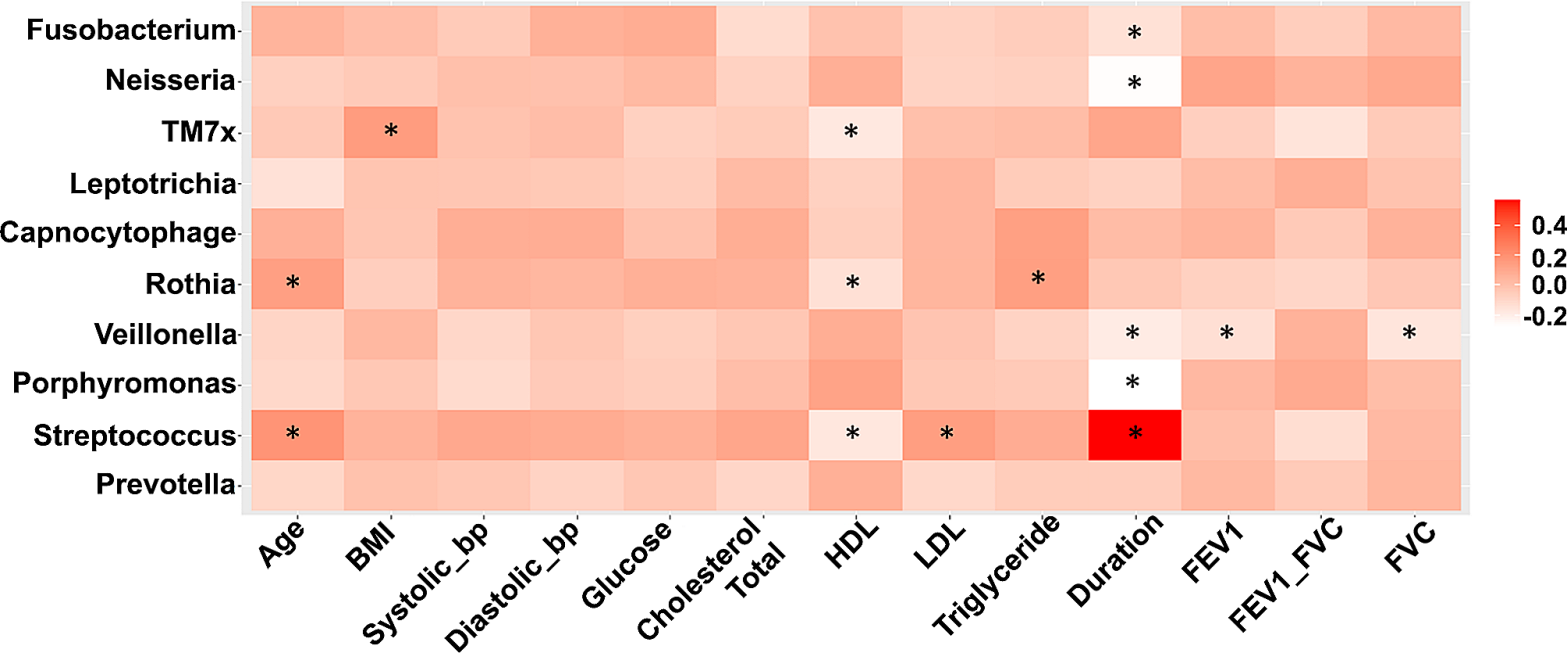
Correlating microbial taxa with participants’ demographic data. Statistical analysis was conducted using Spearman correlation analysis. * p.value < 0.05. Age: years. Duration: Duration of smoking in years. BMI: Body Mass Index (Kg/m^2^). bp: Blood pressure (mm/Hg). FEV1: Forced Expiratory Volume in 1 s. FVC: Forced Vital Capacity. HDL: High density lipoprotein (mmol/L). LDL: Low density lipoprotein (mmol/L). Glucose (mmol/L). Cholesterol Total (mmol/L)

Spearman correlation between the clinical measurements and microbiome taxa is presented in a heatmap in (Fig. 6). The colour intensity (ranging from white and red) indicates the strength of the correlation, r-value, with asterisks indicating significant *p*-values lower than 0.05.

The microbial genera *Streptococcus* was positively correlated with smoking duration (*p* ≤ 0.01; r ≤ + 0.5). However, *Porphyromonas* (*p* ≤ 0.01; *r* ≤ -0.2), *Veillonella* (*p* ≤ 0.05; *r* ≤ -0.2), *Neisseria* (*p* ≤ 0.01; *r* ≤ -0.2), and *Fusobacterium* (*p* ≤ 0.05; *r* ≤ − 0.2) were negatively correlated to smoking duration. HDL was negatively correlated with *TM7x*, *Rothia*, and *Streptococcus*. However, LDL was positively correlated with *Streptococcus*. *Rothia* and *Streptococcus* were positively associated with age, in contrast to *Leptotrichia* that was negatively associated with age. *TM7x* was positively associated with age, and *Rothia* was positively correlated with Triglyceride. Pulmonary function indicators, FEV1 and FVC, were negatively correlated with *Veillonella* and *TM7x* was negatively correlated with FEV1 and FVC.

## Discussion

Smoking has a profound impact on the microbial composition of various body sites, particularly the oral microbiome, which serves as the first point of contact for cigarette smoke toxins. The effects of smoking on microbial ecology include increased saliva acidity, reduced oxygen levels, antibiotic effects, alterations in bacterial adherence to mucosal surfaces, and impaired host immunity [8]. Studies have identified shifts in metabolic pathways, such as decreased aerobic metabolism and increased glycolysis, in smokers [8]. Furthermore, cigarette smoke contains toxins and mutagenic chemicals that can directly interact with human cells in the oral cavity, initiating oral diseases and influencing the cellular environment, including the oral microbiome [22, 23].

Our study aimed to investigate the salivary microbiome composition in smokers and non-smokers within the Qatari population. Analyzing a cohort of 300 Qatari participants, including 200 smokers and 100 non-smokers, we employed sequencing of v3-v4 regions of 16 S rRNA to assess microbial diversity. As expected, smokers exhibited a higher prevalence of harmful bacteria compared to non-smokers, consistent with previous research [4, 10]. Our analysis of alpha diversity metrics, including ASV richness, Shannon, and Simpson index, revealed significantly lower diversity in smokers compared to non-smokers, aligning with prior studies [4, 10]. Beta diversity analysis using Bray-Curtis dissimilarity metrics further underscored significant differences between smoker and non-smoker microbiomes [10].

At the phylum level, smokers exhibited reductions in Proteobacteria and enrichments in Firmicutes and Actinobacteria, which align consistent with metagenomic analyses suggesting changes in oral oxygen availability and breakdown of foreign substances in smokers [8]. Notably, we observed a decrease in Fusobacteriota in smokers, contrary to some previous findings, possibly influenced by dietary factors [4]. Our analysis uncovered variations in genus-level abundance, with smokers displaying decrease in *Neisseria*, *Porphyromonas*, and *Veillonella* compared to non-smokers, suggesting potential shifts in microbial community dynamics linked to smoking [8, 10]. *Streptococcus*, known for its anaerobic characteristics and acid tolerance, was highly abundant in smokers and significantly associated with lipid metabolism markers [8, 24–26].

Age-related changes in saliva composition were evident, with weak positive correlations between age and *Streptococcus* and *Rothia* abundance [28, 29]. Furthermore, we identified associations between certain microbial taxa and health-related biomarkers, such as *TM7x* with BMI and *Rothia* with cardiovascular risk factors, highlighting potential links between oral microbiome composition and systemic health conditions [30, 31].

Limitations of our study include the lack of information on participants’ oral hygiene and oral health, which could potentially influence the observed microbial composition. Additionally, metabolic pathway analysis should be included in future studies to further investigate how the enrichment or depletion of certain bacteria involved in pathways may contribute to disease development. In summary, our study contributes to the growing body of evidence demonstrating the impact of smoking on the salivary microbiome, characterized by dysbiosis favoring anaerobic bacteria. By elucidating these microbial shifts, our findings underscore the importance of smoking cessation interventions in promoting oral and systemic health. The correlations observed between microbial taxa and health biomarkers provide valuable insights into potential mechanisms underlying smoking-related health outcomes. Further research is warranted to explore these associations and their implications for disease prevention and management.

## Conclusion

In conclusion, our study sheds light on smoking-induced dysbiosis in the salivary microbiome and its correlations with lipid biomarkers. These findings underscore the need for targeted interventions to mitigate the adverse effects of smoking on oral and systemic health.

## Data Availability

The data for this study has been deposited in the European Nucleotide Archive (ENA) at EMBL-EBI under the accession number PRJEB73976.

## Abbreviations

COPD: Chronic obstructive pulmonary disease
CVD: Cardiovascular disease
BMI: Body Mass Index
BP: Blood pressure
FEV1: Forced Expiratory Volume in 1 s
FVC: Forced Vital Capacity
HDL: High density lipoprotein
LDL: Low density lipoprotein
RA: Relative Abundance
